# Supplementary material for: Characterisation of the T-cell response to Ebola virus glycoprotein amongst survivors of the 2013–16 West Africa epidemic
Source: Nat Commun. 2021 Feb 19;12:1153. doi: 10.1038/s41467-021-21411-0 (PMC7895930; doi:10.1038/s41467-021-21411-0)
Supplement: Supplementary file 1 — Supplementary Information [file 41467_2021_21411_MOESM1_ESM.pdf]

Supplementary Information

Supplementary figure 1: *EBOV* GP peptide library

This peptide library was ordered from Mimotope. Peptides were resuspended in 10% DMSO to a concentration of 11 mg/ml. Peptides were then pooled and aliquoted. Before use peptides were resuspended to a concentration of 7.5 µg/peptide in L10 media, they were then loaded onto the ELISpot plate, 50 µl/well.

Pep No	Peptide ID	NTerm	Sequence	CTerm	Region	Peptide pool
1	EB-GP-Z-1	H-	MGVTGILQLPRDRFK	-OH	sGP	SP **1-43**
2	EB-GP-Z-2	H-	GILQLPRDRFKRTSF	-OH		
3	EB-GP-Z-3	H-	LPRDRFKRTSFFLWV	-OH		
4	EB-GP-Z-4	H-	RFKRTSFFLWVIILF	-OH		
5	EB-GP-Z-5	H-	TSFFLWVIILFQRTF	-OH		
6	EB-GP-Z-6	H-	LWVIILFQRTFSIPL	-OH		
7	EB-GP-Z-7	H-	ILFQRTFSIPLGVIH	-OH		
8	EB-GP-Z-8	H-	RTFSIPLGVIHNSTL	-OH		
9	EB-GP-Z-9	H-	IPLGVIHNSTLQV	-OH		GP1-1 **33-113**
10	EB-GP-Z-10	H-	LGVIHNSTLQVSDV	-OH		
11	EB-GP-Z-11	H-	IHNSTLQVSDVDKLV	-OH		
12	EB-GP-Z-12	H-	TLQVSDVDKLVCRDK	-OH		
13	EB-GP-Z-13	H-	SDVDKLVCRDKL	-OH		
14	EB-GP-Z-14	H-	DVDKLVCRDKLSSTNQL	-OH		
15	EB-GP-Z-15	H-	CRDKLSSTNQLRSV	-OH		
16	EB-GP-Z-16	H-	KLSSTNQLRSVGLNL	-OH		
17	EB-GP-Z-17	H-	TNQLRSVGLNLEGNGV	-OH		
18	EB-GP-Z-18	H-	SVGLNLEGNGVATDV	-OH		
19	EB-GP-Z-19	H-	NLEGNGVATDVPSA	-OH		
20	EB-GP-Z-20	H-	GNGVATDVPSATKRW	-OH		
21	EB-GP-Z-21	H-	ATDVPSATKRWGFR	-OH		
22	EB-GP-Z-22	H-	VPSATKRWGFRSGV	-OH		
23	EB-GP-Z-23	H-	ATKRWGFRSGVPPKV	-OH		
24	EB-GP-Z-24	H-	WGFRSGVPPKVVNY	-OH		
25	EB-GP-Z-25	H-	RSGVPPKVVNYEA	-OH		
26	EB-GP-Z-26	H-	GVPPKVVNYEAGEWA	-OH		
27	EB-GP-Z-27	H-	KVVNYEAGEWAENCY	-OH		
28	EB-GP-Z-28	H-	YEAGEWAENCYNLEI	-OH		
29	EB-GP-Z-29	H-	EWAENCYNLEIKK	-OH		
30	EB-GP-Z-30	H-	AENCYNLEIKKPDGS	-OH		
31	EB-GP-Z-31	H-	YNLEIKKPDGSECL	-OH		
32	EB-GP-Z-32	H-	EIKKPDGSECLPAA	-OH		

33	EB-GP-Z-33	H-	KPDGSECLPAAPDGI	-OH		
34	EB-GP-Z-34	H-	SECLPAAPDGIRGF	-OH		
35	EB-GP-Z-35	H-	LPAAPDGIRGFPRCR	-OH		
36	EB-GP-Z-36	H-	PDGIRGFPRCRYVHK	-OH		
37	EB-GP-Z-37	H-	RGFPRCRYVHKV	-OH		GP1-2 **103 - 180**
38	EB-GP-Z-38	H-	GFPRCRYVHKVSGTG	-OH		
39	EB-GP-Z-39	H-	CRYVHKVSGTGPCA	-OH		
40	EB-GP-Z-40	H-	VHKVSGTGPCAGDFA	-OH		
41	EB-GP-Z-41	H-	SGTGPCAGDFAFHK	-OH		
42	EB-GP-Z-42	H-	GPCAGDFAFHKEGAF	-OH		
43	EB-GP-Z-43	H-	GDFAFHKEGAFFLY	-OH		
44	EB-GP-Z-44	H-	AFHKEGAFFLYDRLA	-OH		
45	EB-GP-Z-45	H-	EGAFFLYDRLASTVI	-OH		
46	EB-GP-Z-46	H-	FLYDRLASTVIYR	-OH		
47	EB-GP-Z-47	H-	YDRLASTVIYRGTTF	-OH		
48	EB-GP-Z-48	H-	ASTVIYRGTTFAEGV	-OH		
49	EB-GP-Z-49	H-	IYRGTTFAEGVVAFL	-OH		
50	EB-GP-Z-50	H-	TTFAEGVVAFLIL	-OH		
51	EB-GP-Z-51	H-	FAEGVVAFLILPQAK	-OH		
52	EB-GP-Z-52	H-	VVAFLILPQAKKDFF	-OH		
53	EB-GP-Z-53	H-	LILPQAKKDFFSSH	-OH		
54	EB-GP-Z-54	H-	PQAKKDFFSSHPLR	-OH		
55	EB-GP-Z-55	H-	KKDFFSSHPLREPV	-OH		
56	EB-GP-Z-56	H-	FFSSHPLREPVNA	-OH		
57	EB-GP-Z-57	H-	SSHPLREPVNATED	-OH		
58	EB-GP-Z-58	H-	PLREPVNATEDPSSGY	-OH		GP1-3 **170-244**
59	EB-GP-Z-59	H-	VNATEDPSSGYY	-OH		
60	EB-GP-Z-60	H-	NATEDPSSGYYSTTI	-OH		
61	EB-GP-Z-61	H-	DPSSGYYSTTIRYQA	-OH		
62	EB-GP-Z-62	H-	GYYSTTIRYQATGF	-OH		
63	EB-GP-Z-63	H-	STTIRYQATGFGTNE	-OH		
64	EB-GP-Z-64	H-	RYQATGFGTNETEYL	-OH		
65	EB-GP-Z-65	H-	TGFGTNETEYLFEV	-OH		
66	EB-GP-Z-66	H-	GTNETEYLFEVDNL	-OH		
67	EB-GP-Z-67	H-	ETEYLFEVDNLTYV	-OH		
68	EB-GP-Z-68	H-	YLFEVDNLTYVQL	-OH		
69	EB-GP-Z-69	H-	FEVDNLTYVQLESRF	-OH		
70	EB-GP-Z-70	H-	NLTYVQLESRFTPQF	-OH		
71	EB-GP-Z-71	H-	VQLESRFTPQFLLQL	-OH		
72	EB-GP-Z-72	H-	SRFTPQFLLQLNETI	-OH		
73	EB-GP-Z-73	H-	PQFLLQLNETIY	-OH		GP1-4 **234-315**
74	EB-GP-Z-74	H-	FLLQLNETIYTSGKR	-OH		
75	EB-GP-Z-75	H-	LNETIYTSGKRSNTTGK	-OH		
76	EB-GP-Z-76	H-	TSGKRSNTTGKLIWK	-OH		
77	EB-GP-Z-77	H-	RSNTTGKLIWKV	-OH		

78	EB-GP-Z-78	H-	SNTTGKLIWKVNPEI	-OH		
79	EB-GP-Z-79	H-	GKLIWKVNPEIDTTI	-OH		
80	EB-GP-Z-80	H-	WKVNPEIDTTIGEWA	-OH		
81	EB-GP-Z-81	H-	PEIDTTIGEWAFW	-OH		
82	EB-GP-Z-82	H-	IDTTIGEWAFWETKK	-OH		
83	EB-GP-Z-83	H-	IGEWAFWETKKNLTR	-OH		
84	EB-GP-Z-84	H-	AFWETKKNLTRKIR	-OH		
85	EB-GP-Z-85	H-	ETKKNLTRKIRSEEL	-OH		
86	EB-GP-Z-86	H-	NLTRKIRSEELSFTV	-OH		
87	EB-GP-Z-87	H-	KIRSEELSFTVV	-OH		
88	EB-GP-Z-88	H-	IRSEELSFTVVSNGA	-OH		
89	EB-GP-Z-89	H-	ELSFTVVSNGAKNI	-OH		GP1-5a **305-347**
90	EB-GP-Z-90	H-	FTVVSNGAKNISGQSPA	-OH		
91	EB-GP-Z-91	H-	GAKNISGQSPAR	-OH		
92	EB-GP-Z-92	H-	AKNISGQSPARTSSD	-OH		
93	EB-GP-Z-93	H-	SGQSPARTSSDPGTN	-OH		
94	EB-GP-Z-94	H-	PARTSSDPGTNTTTEDH	-OH		
95	EB-GP-Z-95	H-	DPGTNTTTEDHKIMA	-OH		
96	EB-GP-Z-96	H-	NTTTEDHKIMASENSSA	-OH		
97	EB-GP-Z-97	H-	HKIMASENSSAMVQV	-OH	**Exclusive GP**	GP1-5b **341-389**
98	EB-GP-Z-98	H-	ASENSSAMVQVH	-OH		
99	EB-GP-Z-99	H-	SENSSAMVQVHSQGR	-OH		
100	EB-GP-Z-100	H-	SAMVQVHSQGREAAV	-OH		
101	EB-GP-Z-101	H-	VQVHSQGREAAVSHL	-OH		
102	EB-GP-Z-102	H-	SQGREAAVSHLTTLA	-OH		
103	EB-GP-Z-103	H-	EAAVSHLTTLATI	-OH		
104	EB-GP-Z-104	H-	AVSHLTTLATISTS	-OH		
105	EB-GP-Z-105	H-	HLTTLATISTSPQSL	-OH		
106	EB-GP-Z-106	H-	LATISTSPQSLTTK	-OH		
107	EB-GP-Z-107	H-	ISTSPQSLTTKPGPD	-OH		
108	EB-GP-Z-108	H-	PQSLTTKPGPDNSTH	-OH		
109	EB-GP-Z-109	H-	TTKPGPDNSTHNTPV	-OH		GP1-6 **387-459**
110	EB-GP-Z-110	H-	GPDNSTHNTPVYKL	-OH		
111	EB-GP-Z-111	H-	NSTHNTPVYKLDI	-OH		
112	EB-GP-Z-112	H-	THNTPVYKLDISEA	-OH		
113	EB-GP-Z-113	H-	TPVYKLDISEATQV	-OH		
114	EB-GP-Z-114	H-	YKLDISEATQVEQHH	-OH		
115	EB-GP-Z-115	H-	ISEATQVEQHHRR	-OH		
116	EB-GP-Z-116	H-	EATQVEQHHRRTDND	-OH		
117	EB-GP-Z-117	H-	VEQHHRRTDNDSTA	-OH		
118	EB-GP-Z-118	H-	HHRRTDNDSTASDTPSA	-OH		
119	EB-GP-Z-119	H-	NDSTASDTPSATTAA	-OH		
120	EB-GP-Z-120	H-	ASDTPSATTAAGPPK	-OH		
121	EB-GP-Z-121	H-	PSATTAAGPPKA	-OH		
122	EB-GP-Z-122	H-	SATTAAGPPKAENTN	-OH		

123	EB-GP-Z-123	H-	AAGPPKAENTNTSK	-OH		
124	EB-GP-Z-124	H-	PPKAENTNTSKSTDF	-OH		
125	EB-GP-Z-125	H-	ENTNTSKSTDFLDPA	-OH		
126	EB-GP-Z-126	H-	TSKSTDFLDPATTTS	-OH		
127	EB-GP-Z-127	H-	TDFLDPATTTSPQNH	-OH		
128	EB-GP-Z-128	H-	DPATTTSPQNHSETA	-OH		
129	EB-GP-Z-129	H-	TTSPQNHSETAGNNNTH	-OH		
130	EB-GP-Z-130	H-	HSETAGNNNTHH	-OH		
131	EB-GP-Z-131	H-	SETAGNNNTHHQDTG	-OH		
132	EB-GP-Z-132	H-	GNNNTHHQDTGEESA	-OH		
133	EB-GP-Z-133	H-	THHQDTGEESASSGK	-OH		
134	EB-GP-Z-134	H-	DTGEESASSGKLGLI	-OH		
135	EB-GP-Z-135	H-	ESASSGKLGLITNTI	-OH		
136	EB-GP-Z-136	H-	SGKLGLITNTIAGVA	-OH		
137	EB-GP-Z-137	H-	GLITNTIAGVAGLI	-OH		GP1-7 **449-525**
138	EB-GP-Z-138	H-	TNTIAGVAGLITGGR	-OH		
139	EB-GP-Z-139	H-	AGVAGLITGGRRTRR	-OH		
140	EB-GP-Z-140	H-	GLITGGRRTRREAIV	-OH		
141	EB-GP-Z-141	H-	GGRRTRREAIVNA	-OH		
142	EB-GP-Z-142	H-	RRTRREAIVNAQPK	-OH		
143	EB-GP-Z-143	H-	RREAIVNAQPKCNPNL	-OH		
144	EB-GP-Z-144	H-	VNAQPKCNPNLHYW	-OH		
145	EB-GP-Z-145	H-	AQPKCNPNLHYW	-OH		
146	EB-GP-Z-146	H-	PKCNPNLHYWTTQDEGA	-OH		
147	EB-GP-Z-147	H-	LHYWTTQDEGAAIGL	-OH		
148	EB-GP-Z-148	H-	TTQDEGAAIGLAWI	-OH		
149	EB-GP-Z-149	H-	DEGAAIGLAWIPYF	-OH		
150	EB-GP-Z-150	H-	AAIGLAWIPYFGPAA	-OH		
151	EB-GP-Z-151	H-	LAWIPYFGPAAEGIY	-OH		
152	EB-GP-Z-152	H-	PYFGPAAEGIYIEGL	-OH		
153	EB-GP-Z-153	H-	PAAEGIYIEGLMH	-OH		
154	EB-GP-Z-154	H-	AEGIYIEGLMHNQDGL	-OH		
155	EB-GP-Z-155	H-	IEGLMHNQDGLICGL	-OH		
156	EB-GP-Z-156	H-	MHNQDGLICGLRQLA	-OH		GP2-1 **517-602**
157	EB-GP-Z-157	H-	DGLICGLRQLANETTQA	-OH		
158	EB-GP-Z-158	H-	LRQLANETTQALQLF	-OH		
159	EB-GP-Z-159	H-	ANETTQALQLFLRA	-OH		
160	EB-GP-Z-160	H-	TTQALQLFLRATTEL	-OH		
161	EB-GP-Z-161	H-	LQLFLRATTELRTF	-OH		
162	EB-GP-Z-162	H-	FLRATTELRTFSIL	-OH		
163	EB-GP-Z-163	H-	ATTELRTFSILNRKA	-OH		
164	EB-GP-Z-164	H-	LRTFSILNRKAIDFL	-OH		
165	EB-GP-Z-165	H-	SILNRKAIDFLLQRW	-OH		
166	EB-GP-Z-166	H-	RKAIDFLLQRWGGTCH	-OH		
167	EB-GP-Z-167	H-	FLLQRWGGTCHIL	-OH		

168	EB-GP-Z-168	H-	LQRWGGTCHILGPDCCI	-OH		GP2-2 **594-676**
169	EB-GP-Z-169	H-	TCHILGPDCCIEPH	-OH		
170	EB-GP-Z-170	H-	ILGPDCCIEPHDWTK	-OH		
171	EB-GP-Z-171	H-	DCCIEPHDWTKNI	-OH		
172	EB-GP-Z-172	H-	CIEPHDWTKNITDKI	-OH		
173	EB-GP-Z-173	H-	HDWTKNITDKIDQII	-OH		
174	EB-GP-Z-174	H-	KNITDKIDQIIHDFV	-OH		
175	EB-GP-Z-175	H-	DKIDQIIHDFVDKTL	-OH		
176	EB-GP-Z-176	H-	DQIIHDFVDKTL	-OH		
177	EB-GP-Z-177	H-	IIHDFVDKTLPDQGD	-OH		
178	EB-GP-Z-178	H-	FVDKTLPDQGDNDNW	-OH		
179	EB-GP-Z-179	H-	TLPDQGDNDNWWTGW	-OH		
180	EB-GP-Z-180	H-	DQGDNDNWWTGWRQW	-OH		
181	EB-GP-Z-181	H-	NDNWWTGWRQWIPA	-OH		
182	EB-GP-Z-182	H-	WWTGWRQWIPAGIGV	-OH		
183	EB-GP-Z-183	H-	WRQWIPAGIGVTGVV	-OH		
184	EB-GP-Z-184	H-	IPAGIGVTGVVIAVI	-OH		
185	EB-GP-Z-185	H-	IGVTGVVIAVIALF	-OH		
186	EB-GP-Z-186	H-	TGVVIAVIALFCICK	-OH		
187	EB-GP-Z-187	H-	IAVIALFCICKFVF	-OH		

Supplementary figure 2: Correlations between IFNγ ELISpot and humoral immune response

Correlation between the total summed ELISpot response and matched humoral response. **A**) whole virus ELISA IgG response. **B**) Live virus neutralisation response and **C**) anti-GP IgG response.

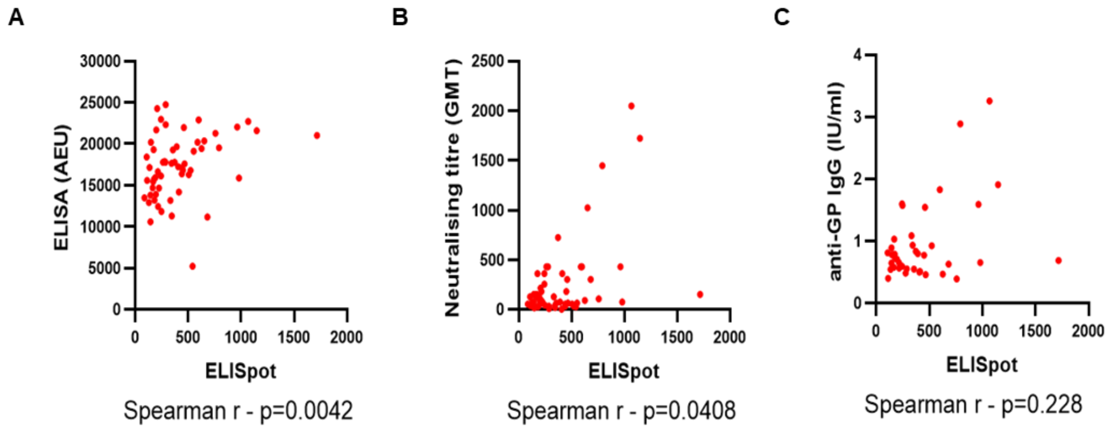

Supplementary figure 3: Individual ELISpot response to GP peptide pools

IFNγ ELISpot using EVD survivor PBMC stimulated with *EBOV* GP peptide pools. The individual response by each survivor can be seen in the density plot with the darker squares representing a higher SFU/10^6 cells.

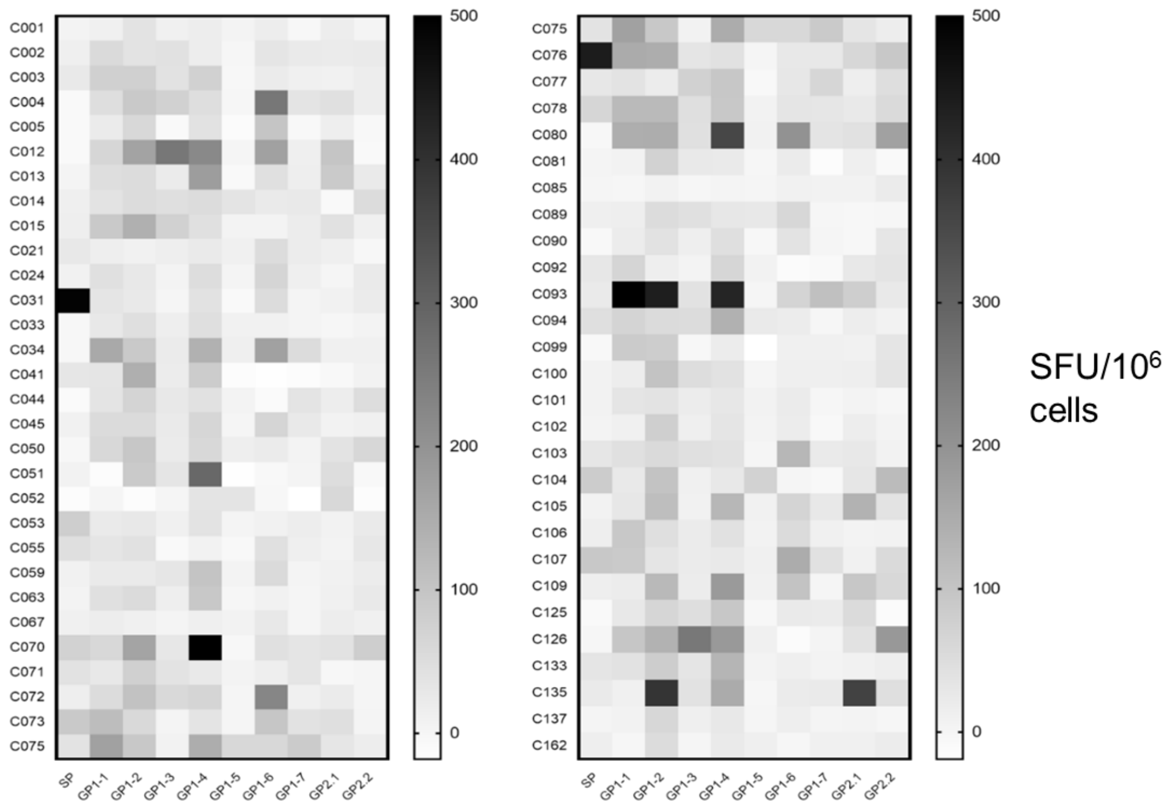

Supplementary figure 4: Individual ELISpot response to all GP peptides

IFNγ ELISpot response amongst 15 EVD survivors to each individual peptide that contributes to our *EBOV* peptide library. Data was acquired on fresh PBMC in the field, n=1 each bar graph represents the results from a single EVD survivor a single well of an ELISpot plate relates to a single peptide in our GP library.

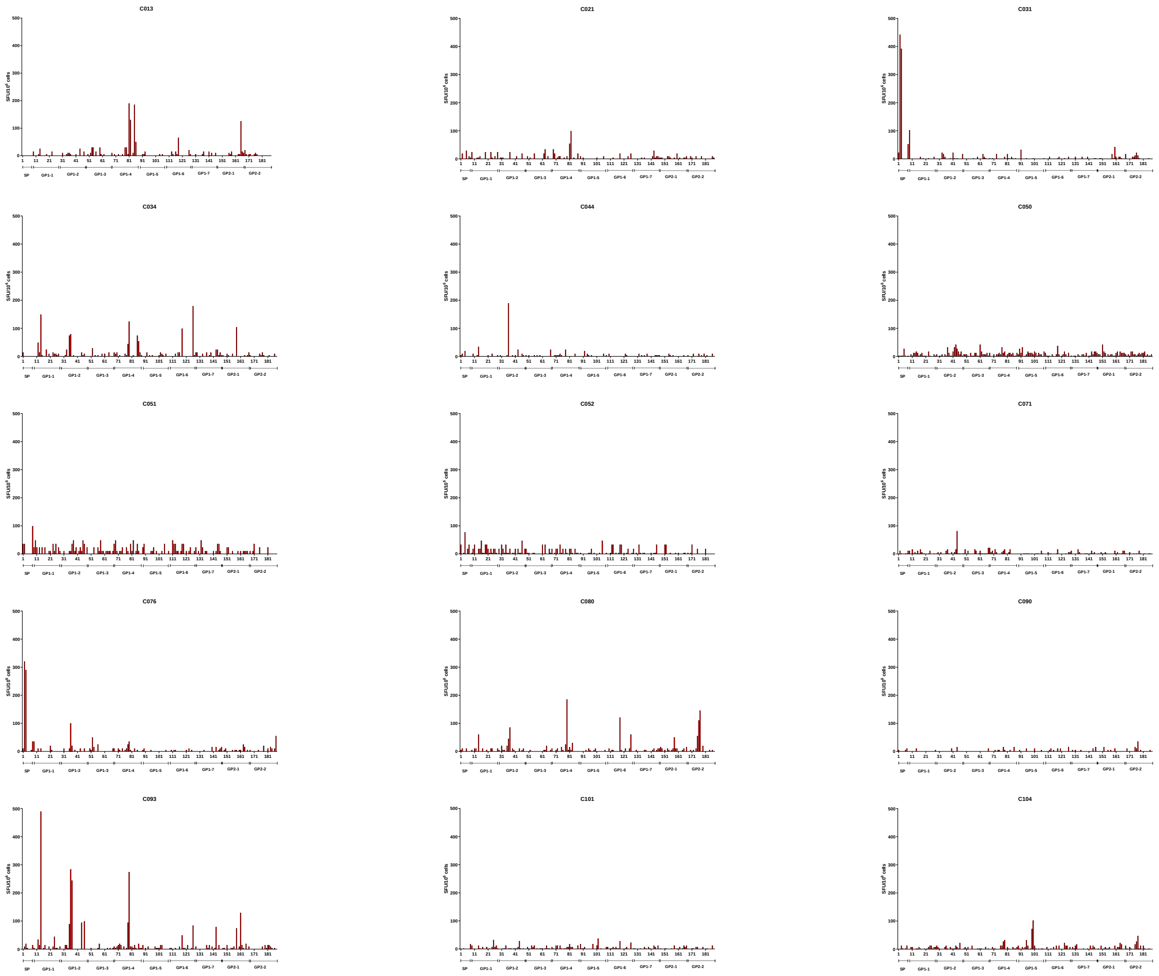

Supplementary figure 5: Flow cytometry gating strategy

Samples were collected in triplicate on a 96-well plate using a BD Fortessa machine. **A**) Samples are displayed by forward and side scatter and the lymphocyte region gated. This is then investigated for doublets before being displayed as BV510 (dead) by APC Alexa 750 (CD3) the live CD3 cells were exported and concatenated with their corresponding triplicate to give a new FCS file. This was to reduce the size of the file for downstream analysis. **B**) Samples were next displayed as BV786 (CD4) by AF700 (CD8) and either the CD4 or CD8 positive population gated. Both CD4 or CD8 populations were displayed as APC (CCR7) by BV605 (CD45RO) which gave a broad view of the cell phenotype. Both CD4 or CD8 populations were displayed as CCR7 by either PE (IL-2), BV410 (TNFα), Alexa 488 (IFNγ) or PerCP cy 5.5 (CD107a – CD8 only) to give information on the cytokine and activation response.

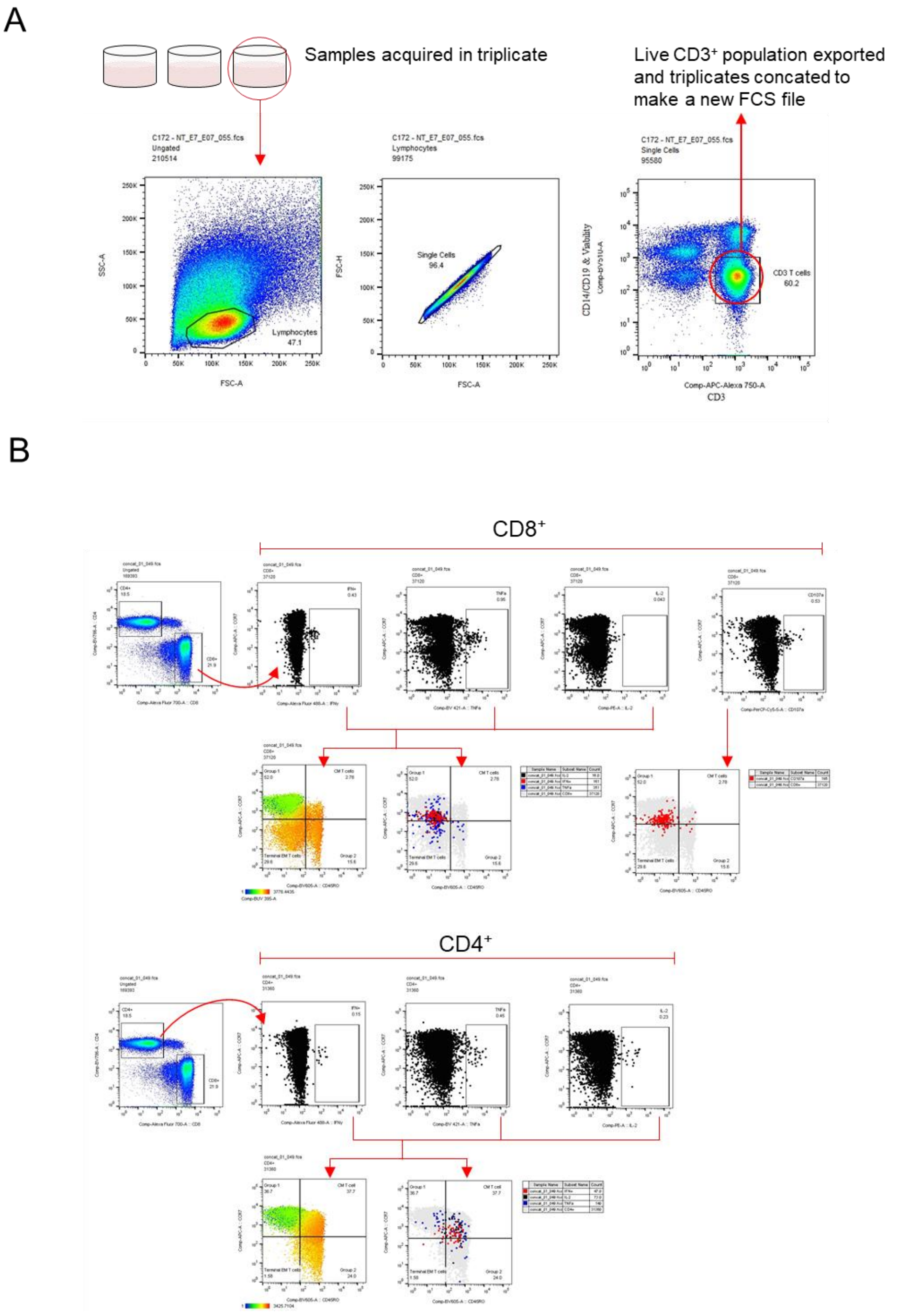

Supplementary figure 6: CD28 and CD95 expression on GP peptide library specific CD8+ T cells.

EVD survivor C070 whose PBMC were stimulated overnight with GP peptide pool. CD8^{+} T cells producing IFNγ and TNFα in response are overlaid on top of the CD28 vs CD95 dot plot, these cells have originated from the group 1 gate.

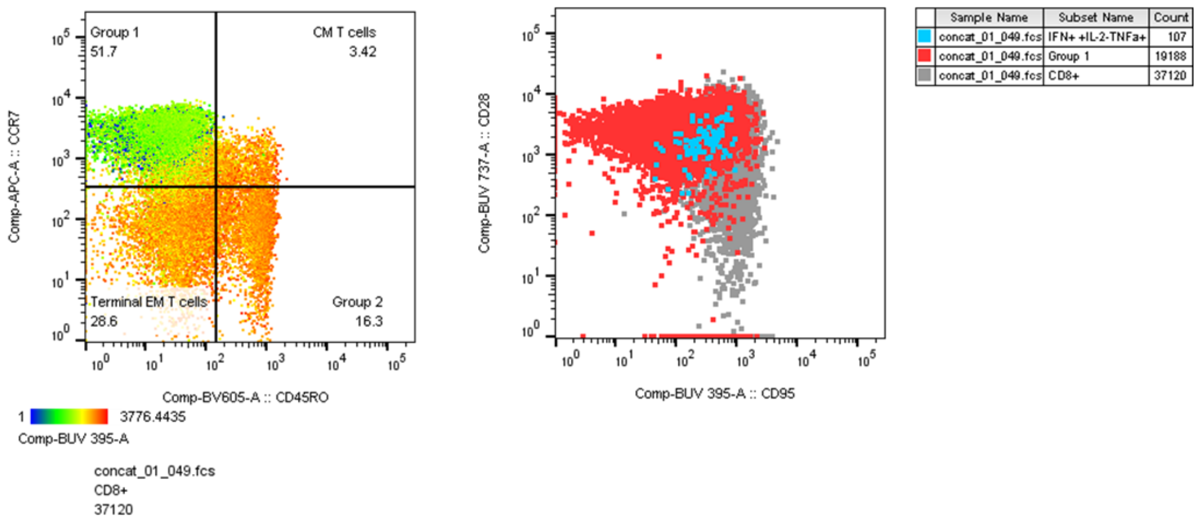

Supplementary figure 7: CD107a staining following overnight stimulation with GP peptide library (187 peptides)

Survivor or negative PBMC samples were stimulated overnight with GP peptide library. Red or blue dots represent individual data points and the bars show the median values plus 95% CI. No significant difference was found, significance was looked for using two tailed Mann-Whitney U test p=0.35, n=32 EVD survivors and 18 EVD negative samples.

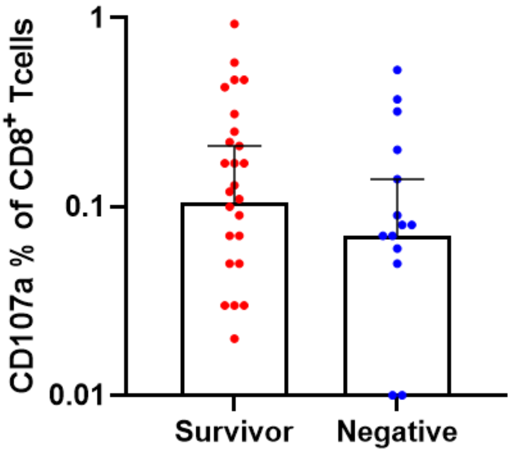

Supplementary figure 8: ICS assays looking at peptide 3 and GP1-2 peptide stimulation

Intracellular cytokine staining experiments using either **A**) peptide 3 (LPRDRFKRTSFFLWV) amongst 8 EVD survivors who had previously responded to peptide pool SP or **B**) GP1-2 peptide sub pools. Within B peptide GP1-2 sub pool 1 corresponds to peptides 29-33, GP1-2 sub pool 2 corresponds to peptides 34-40 and GP1-2 sub pool 3 corresponds to peptides 41-48. N= 4 EVD survivors and one EVD negative. Error bars represent the mean +SD.

A

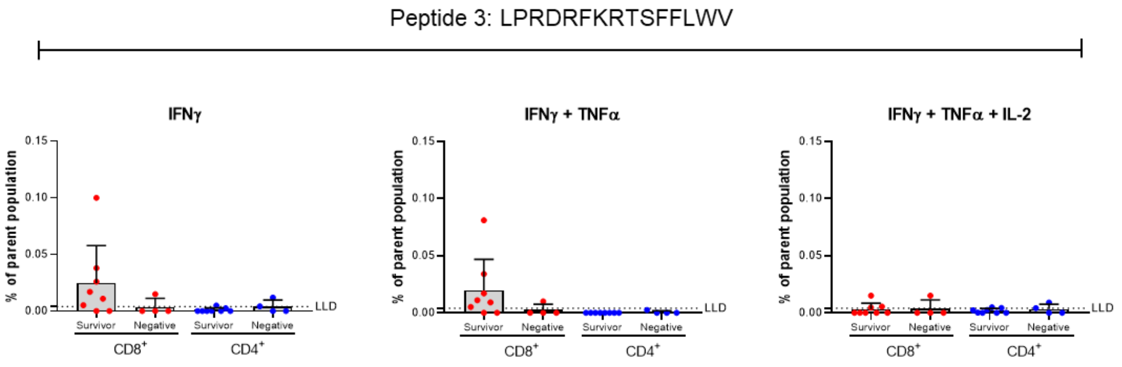

B

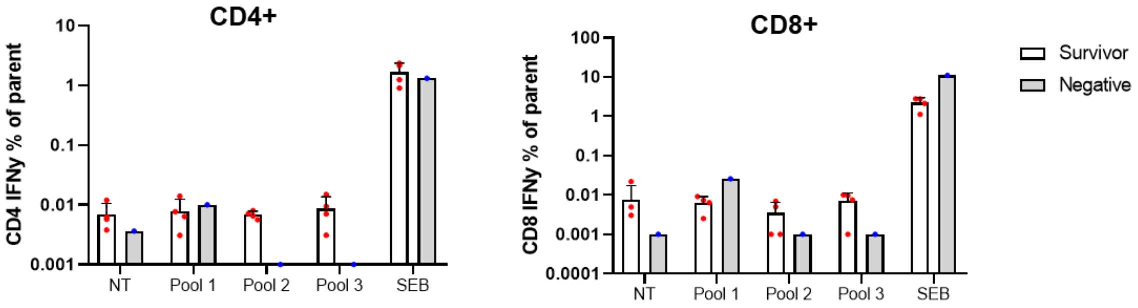

Supplementary figure 9: MHCI and MHCII frequency within our survivor cohort - the top 10 most common alleles only are displayed.

MHCI

HLA-A	No	Freq	HLA-B	No	Freq	HLA-C	No	Freq
02:01	22	0.29	53:01	23	0.31	04:01	29	0.39
23:01	21	0.28	35:01	21	0.28	16:01	29	0.39
30:01	16	0.21	42:01	13	0.17	07:01	18	0.24
30:02	10	0.13	15:03	12	0.16	17:01	14	0.19
02:02	10	0.13	78:01	10	0.13	02:10	11	0.15
33:03	9	0.12	58:01	10	0.13	06:02	10	0.13
34:02	8	0.11	07:02	8	0.11	03:02	8	0.11
68:02	8	0.11	08:01	6	0.08	03:04	6	0.08
33:01	7	0.09	45:01	6	0.08	07:02	6	0.08
74:01	7	0.09	18:01	6	0.08	01:02	5	0.07

MHCII

DPA	No	Freq	DPB	No	Freq	DQA	No	Freq	DQB	No	Freq	DRB	No	Freq
02:01	62	0.83	01:01	62	0.83	05:05	38	0.51	03:19	31	0.41	03:02	26	0.35
02:02	32	0.43	17:01	15	0.20	01:02	33	0.44	04:02	26	0.35	11:01	13	0.17
03:01	29	0.39	105:01	15	0.20	04:01	28	0.37	02:02	21	0.28	13:04	12	0.16
01:03	18	0.24	02:01	10	0.13	03:03	19	0.25	05:01	15	0.20	13:02	11	0.15
02:07	9	0.12	13:01	10	0.13	02:01	9	0.12	06:02	13	0.17	09:01	11	0.15
			85:01	9	0.12	01:01	8	0.11	06:03	9	0.12	11:02	9	0.12
			04:01	8	0.11	01:03	7	0.09	03:01	8	0.11	13:01	9	0.12
			131:01	7	0.09	05:01	5	0.07	03:02	7	0.09	04:05	8	0.11
			39:01	3	0.04	01:05	1	0.01	06:09	6	0.08	07:01	8	0.11
			350:01	2	0.03	05:02	1	0.01	05:02	6	0.08	08:06	7	0.09
