# Supplementary material for: Characterisation of the T-cell response to Ebola virus glycoprotein amongst survivors of the 2013–16 West Africa epidemic
Source: Nat Commun. 2021 Feb 19;12:1153. doi: 10.1038/s41467-021-21411-0 (PMC7895930; doi:10.1038/s41467-021-21411-0)
Supplement: Supplementary file 3 — Reporting Summary [file 41467_2021_21411_MOESM3_ESM.pdf]

nature research

Corresponding author(s): Tom Tipton

Last updated by author(s): Nov 20, 2020

Reporting Summary

Nature Research wishes to improve the reproducibility of the work that we publish. This form provides structure for consistency and transparency in reporting. For further information on Nature Research policies, see our Editorial Policies and the Editorial Policy Checklist.

Statistics

For all statistical analyses, confirm that the following items are present in the figure legend, table legend, main text, or Methods section.

n/a	Confirmed	
☐	☒	The exact sample size (*n*) for each experimental group/condition, given as a discrete number and unit of measurement
☐	☒	A statement on whether measurements were taken from distinct samples or whether the same sample was measured repeatedly
☐	☒	The statistical test(s) used AND whether they are one- or two-sided<br>*Only common tests should be described solely by name; describe more complex techniques in the Methods section.*
☐	☒	A description of all covariates tested
☒	☐	A description of any assumptions or corrections, such as tests of normality and adjustment for multiple comparisons
☐	☒	A full description of the statistical parameters including central tendency (e.g. means) or other basic estimates (e.g. regression coefficient) AND variation (e.g. standard deviation) or associated estimates of uncertainty (e.g. confidence intervals)
☐	☒	For null hypothesis testing, the test statistic (e.g. *F*, *t*, *r*) with confidence intervals, effect sizes, degrees of freedom and *P* value noted<br>*Give P values as exact values whenever suitable.*
☒	☐	For Bayesian analysis, information on the choice of priors and Markov chain Monte Carlo settings
☒	☐	For hierarchical and complex designs, identification of the appropriate level for tests and full reporting of outcomes
☒	☐	Estimates of effect sizes (e.g. Cohen's *d*, Pearson's *r*), indicating how they were calculated

Our web collection on statistics for biologists contains articles on many of the points above.

Software and code

Policy information about availability of computer code

Data collection	FACS DIVA, Omixon HLA Explore (beta version)
Data analysis	FlowJo v10, IEDB.org, GraphPad v8.

For manuscripts utilizing custom algorithms or software that are central to the research but not yet described in published literature, software must be made available to editors and reviewers. We strongly encourage code deposition in a community repository (e.g. GitHub). See the Nature Research guidelines for submitting code & software for further information.

Data

Policy information about availability of data

All manuscripts must include a data availability statement. This statement should provide the following information, where applicable:

- Accession codes, unique identifiers, or web links for publicly available datasets
- A list of figures that have associated raw data
- A description of any restrictions on data availability

Mimotopes GP library based on UniProtKB - Q05320 (VGP_EBOZM).https://www.uniprot.org/uniprot/Q05320

Field-specific reporting

Life sciences study design

All studies must disclose on these points even when the disclosure is negative.

Sample size	No sample size calculation was performed as this was an observational cohort study, survivors of Ebola virus disease were contacted by local survivor group leaders and if consenting were enrolled onto the study.
Data exclusions	No data exclusions
Replication	ELISpot samples were acquired in duplicate or triplicate, replications were successful. For flow cytometry studies a non stimulated and SEB stimulated control were run, due to the precious nature of the PBMC samples multiple repeats of these experiment were not possible.
Randomization	Participants were assigned into Ebola survivor or negative groups based on whether they received a PCR positive Ebola diagnosis during the 2013-16 Ebola epidemic. Other co variates were not controlled
Blinding	There was no blinding of samples in this study.

Reporting for specific materials, systems and methods

We require information from authors about some types of materials, experimental systems and methods used in many studies. Here, indicate whether each material, system or method listed is relevant to your study. If you are not sure if a list item applies to your research, read the appropriate section before selecting a response.

Materials & experimental systems

n/a	Involved in the study	
☐	☒	Antibodies
☒	☐	Eukaryotic cell lines
☒	☐	Palaeontology and archaeology
☒	☐	Animals and other organisms
☐	☒	Human research participants
☒	☐	Clinical data
☒	☐	Dual use research of concern

Methods

n/a	Involved in the study	
☒	☐	ChIP-seq
☐	☒	Flow cytometry
☒	☐	MRI-based neuroimaging

Antibodies

Antibodies used

Anti-Human CD107a PerCP cy5.5 Mouse IgG1, κ H4A3 328616 Biolegend
Anti-Human IFN-g Alexa 488 IgG1 Kappa 4S.B3 502515 Biolegend
Anti-Human CD4 BV 785 Mouse IgG2b, κ OKT4 317442 Biolegend
Anti-HumanCD45RO BV 605 Mouse IgG2a, κ UCHL1 304238 Biolegend
Anti-Human CD14 BV 510 Mouse IgG2a, κ M5E2 301842 Biolegend
Anti-Human CD19 BV 510 Mouse IgG1, κ HIB19 302242 Biolegend
Anti-Human TNFalpha BV 421 Mouse IgG1, κ mAb 11 502932 Biolegend
Anti-Human CD3 APC fire 750 Mouse IgG1, κ SK7 344840 Biolegend
Anti-Human CD8a Alexa flour 700 Mouse IgG1, κ HIT8a 300920 Biolegend
Anti-Human CCR7 APC Mouse IgG2a, κ G043H7 353214 Biolegend
Anti-Human IL-2 PE Rat IgG2a, κ MQ1-17H12 500307 Biolegend
CD49d Purified Mouse IgG1, κ 9F10 304302 Biolegend
Anti-Human CED28 (BD) BUV 737 Mouse IgG1, κ CD28.2 564438 BD
Anti-Human CD95 BUV 395 Mouse IgG1, κ DX2 740306 BD

Validation

Anti-Human CD107a PerCP cy5.5 Mouse IgG1, κ H4A3 328616 Biolegend https://www.biolegend.com/en-us/products/percp-cyanine5-5-anti-human-cd107a-lamp-1-antibody-5365

Anti-Human IFN-g Alexa 488 IgG1 Kappa 4S.B3 502515 Biolegend https://www.biolegend.com/en-us/products/alexa-fluor-488-anti-human-ifn-gamma-antibody-2748

Anti-Human CD4 BV 785 Mouse IgG2b, κ OKT4 317442 Biolegend https://www.biolegend.com/en-us/products/brilliant-violet-785-anti-human-cd4-antibody-7978

Anti-HumanCD45RO BV 605 Mouse IgG2a, κ UCHL1 304238 Biolegend https://www.biolegend.com/en-us/products/brilliant-violet-605-anti-human-cd45ro-antibody-8569

Anti-Human CD14 BV 510 Mouse IgG2a, κ M5E2 301842 Biolegend https://www.biolegend.com/en-us/products/brilliant-

violet-510-anti-human-cd14-antibody-8001

Anti-Human CD19 BV 510 Mouse IgG1, κ HIB19 302242 Biolegend https://www.biolegend.com/en-us/products/brilliant-violet-510-anti-human-cd19-antibody-8004

Anti-Human TNFalpha BV 421 Mouse IgG1, κ mAb 11 502932 Biolegend https://www.biolegend.com/en-us/products/brilliant-violet-421-anti-human-tnf-alpha-antibody-7215

Anti-Human CD3 APC fire 750 Mouse IgG1, κ SK7 344840 Biolegend https://www.biolegend.com/en-us/products/apc-fire-750-anti-human-cd3-antibody-13004

Anti-Human CD8a Alexa flour 700 Mouse IgG1, κ HIT8a 300920 Biolegend https://www.biolegend.com/en-us/products/alexa-fluor-700-anti-human-cd8a-antibody-3434

Anti-Human CCR7 APC Mouse IgG2a, κ G043H7 353214 Biolegend https://www.biolegend.com/en-us/products/apc-anti-human-cd197-ccr7-antibody-7536

Anti-Human IL-2 PE Rat IgG2a, κ MQ1-17H12 500307 Biolegend https://www.biolegend.com/en-us/products/pe-anti-human-il-2-antibody-1351

CD49d Purified Mouse IgG1, κ 9F10 304302 Biolegend https://www.biolegend.com/en-us/products/purified-anti-human-cd49d-antibody-586

Anti-Human CED28 (BD) BUV 737 Mouse IgG1, κ CD28.2 564438 BD https://www.bdbiosciences.com/ds/pm/tds/564438.pdf

Anti-Human CD95 BUV 395 Mouse IgG1, κ DX2 740306 BD https://www.bdbiosciences.com/eu/reagents/research/antibodies-buffers/immunology-reagents/anti-human-antibodies/cell-surface-antigens/buv395-mouse-anti-human-cd95-dx2/p/740306

Human research participants

Policy information about studies involving human research participants

Population characteristics	All participants were from Guinea and aged over 16, a mix of males and females participated. We have no information on past or current diagnosis and treatment categories.
Recruitment	Participants were members of an Ebola survivors association and were recruited in collaboration with the Ebola survivor association leadership. We know of no potential self selection bias that may have been present.
Ethics oversight	Ethical approval was obtained from the National Ethics Committee for Health Research, Guinea (No. 33/CNERS/15) and from the National Research Ethics Service, UK.

Note that full information on the approval of the study protocol must also be provided in the manuscript.

Flow Cytometry

Plots

Confirm that:

- [x] The axis labels state the marker and fluorochrome used (e.g. CD4-FITC).
- [x] The axis scales are clearly visible. Include numbers along axes only for bottom left plot of group (a 'group' is an analysis of identical markers).
- [x] All plots are contour plots with outliers or pseudocolor plots.
- [x] A numerical value for number of cells or percentage (with statistics) is provided.

Methodology

Sample preparation	Human PBMC processed from peripheral blood
Instrument	Fortessa II special order machine
Software	Data collected using FACS diva, data analysed using FlowJo v10
Cell population abundance	cells were not sorted and reflect the percentage of their parent population.
Gating strategy	FSC/SSC were used to gate on lymphosytes, FSC hight vs width was used to exclude doublets, Live CD3+ cells were gated upon then either CD4 or CD8+ cells were gated. Within each of these populatuion IFN, TNF, IL2 or CD107a were gated following relevant stimulations. CD4 or CD8 cells were viewed by CCR7 vs CD45RO.

- [x] Tick this box to confirm that a figure exemplifying the gating strategy is provided in the Supplementary Information.
